# Concurrent once-daily versus twice-daily chemoradiotherapy in patients with limited-stage small-cell lung cancer (CONVERT): an open-label, phase 3, randomised, superiority trial

**DOI:** 10.1016/S1470-2045(17)30318-2

**Published:** 2017-08

**Authors:** Corinne Faivre-Finn, Michael Snee, Linda Ashcroft, Wiebke Appel, Fabrice Barlesi, Adityanarayan Bhatnagar, Andrea Bezjak, Felipe Cardenal, Pierre Fournel, Susan Harden, Cecile Le Pechoux, Rhona McMenemin, Nazia Mohammed, Mary O'Brien, Jason Pantarotto, Veerle Surmont, Jan P Van Meerbeeck, Penella J Woll, Paul Lorigan, Fiona Blackhall

**Affiliations:** a Division of Molecular and Clinical Cancer Sciences, School of Medical Sciences, Faculty of Biology, Medicine and Health, University of Manchester, Manchester, UK; b Department of Radiotherapy Related Research, The Christie NHS Foundation Trust, Manchester, UK; c St James Institute of Oncology, Leeds, UK; d Manchester Academic Health Science Centre Trials Co-ordination Unit, The Christie NHS Foundation Trust, Manchester, UK; e Rosemere Cancer Centre, Lancashire Teaching Hospitals NHS Foundation Trust, Preston, UK; f Multidisciplinary Oncology & Therapeutic Innovations Department, Aix Marseille Univ, Assistance Publique Hôpitaux de Marseille, Marseille, France; g Department of Clinical Oncology, University Hospital Southampton NHS Foundation Trust, Southampton, UK; h Canadian Cancer Trials Group, Princess Margaret Cancer Center, Toronto, ON, Canada; i GECP, Department of Medical Oncology, Institut Català'Oncologia, L'Hospitalet (Barcelona), Barcelona, Spain; j GFPC, Département d'Oncologie Médicale, Institut de Cancérologie Lucien Neuwirth, Saint-Étienne, France; k Department of Oncology, Cambridge University Hospitals NHS Foundation Trust, Cambridge, UK; l Département d'Oncologie Radiothérapie, Gustave Roussy Cancer Campus, Villejuif, France; m Northern Centre for Cancer Care, Newcastle Hospitals NHS Foundation Trust, Newcastle upon Tyne, UK; n Beatson West of Scotland Cancer Centre, NHS Greater Glasgow and Clyde, Glasgow, UK; o Department of Medicine, Royal Marsden NHS Foundation Trust, Surrey, UK; p Division of Radiation Oncology, University of Ottawa, Ottawa, ON, Canada; q Department of Respiratory Medicine/Thoracic Oncology, Ghent University Hospital, Ghent, Belgium; r Thoracic Oncology, Antwerp University Hospital, Antwerp, Belgium; s Department of Oncology & Metabolism, University of Sheffield, Sheffield, UK

## Abstract

**Background:**

Concurrent chemoradiotherapy is the standard of care in limited-stage small-cell lung cancer, but the optimal radiotherapy schedule and dose remains controversial. The aim of this study was to establish a standard chemoradiotherapy treatment regimen in limited-stage small-cell lung cancer.

**Methods:**

The CONVERT trial was an open-label, phase 3, randomised superiority trial. We enrolled adult patients (aged ≥18 years) who had cytologically or histologically confirmed limited-stage small-cell lung cancer, Eastern Cooperative Oncology Group performance status of 0–2, and adequate pulmonary function. Patients were recruited from 73 centres in eight countries. Patients were randomly assigned to receive either 45 Gy radiotherapy in 30 twice-daily fractions of 1·5 Gy over 19 days, or 66 Gy in 33 once-daily fractions of 2 Gy over 45 days, starting on day 22 after commencing cisplatin–etoposide chemotherapy (given as four to six cycles every 3 weeks in both groups). The allocation method used was minimisation with a random element, stratified by institution, planned number of chemotherapy cycles, and performance status. Treatment group assignments were not masked. The primary endpoint was overall survival, defined as time from randomisation until death from any cause, analysed by modified intention-to-treat. A 12% higher overall survival at 2 years in the once-daily group versus the twice-daily group was considered to be clinically significant to show superiority of the once-daily regimen. The study is registered with ClinicalTrials.gov (NCT00433563) and is currently in follow-up.

**Findings:**

Between April 7, 2008, and Nov 29, 2013, 547 patients were enrolled and randomly assigned to receive twice-daily concurrent chemoradiotherapy (274 patients) or once-daily concurrent chemoradiotherapy (273 patients). Four patients (one in the twice-daily group and three in the once-daily group) did not return their case report forms and were lost to follow-up; these patients were not included in our analyses. At a median follow-up of 45 months (IQR 35–58), median overall survival was 30 months (95% CI 24–34) in the twice-daily group versus 25 months (21–31) in the once-daily group (hazard ratio for death in the once daily group 1·18 [95% CI 0·95–1·45]; p=0·14). 2-year overall survival was 56% (95% CI 50–62) in the twice-daily group and 51% (45–57) in the once-daily group (absolute difference between the treatment groups 5·3% [95% CI −3·2% to 13·7%]). The most common grade 3–4 adverse event in patients evaluated for chemotherapy toxicity was neutropenia (197 [74%] of 266 patients in the twice-daily group *vs* 170 [65%] of 263 in the once-daily group). Most toxicities were similar between the groups, except there was significantly more grade 4 neutropenia with twice-daily radiotherapy (129 [49%] *vs* 101 [38%]; p=0·05). In patients assessed for radiotherapy toxicity, was no difference in grade 3–4 oesophagitis between the groups (47 [19%] of 254 patients in the twice-daily group *vs* 47 [19%] of 246 in the once-daily group; p=0·85) and grade 3–4 radiation pneumonitis (4 [3%] of 254 *vs* 4 [2%] of 246; p=0·70). 11 patients died from treatment-related causes (three in the twice-daily group and eight in the once-daily group).

**Interpretation:**

Survival outcomes did not differ between twice-daily and once-daily concurrent chemoradiotherapy in patients with limited-stage small-cell lung cancer, and toxicity was similar and lower than expected with both regimens. Since the trial was designed to show superiority of once-daily radiotherapy and was not powered to show equivalence, the implication is that twice-daily radiotherapy should continue to be considered the standard of care in this setting.

**Funding:**

Cancer Research UK (Clinical Trials Awards and Advisory Committee), French Ministry of Health, Canadian Cancer Society Research Institute, European Organisation for Research and Treatment of Cancer (Cancer Research Fund, Lung Cancer, and Radiation Oncology Groups).

**Research in context**

**Evidence before this study**

The role of thoracic radiotherapy is well established in the management of limited-stage small-cell lung cancer, and the standard of care in patients with good performance status is concurrent chemoradiotherapy. However, the optimal radiotherapy dose and fractionation remains controversial. One standard of care is twice-daily radiotherapy, which was shown to be superior to once-daily radiotherapy in a landmark Intergroup 0096 study in 1999. We searched PubMed and the abstracts of major conferences (such as the American Society of Clinical Oncology) with the terms “small cell lung cancer”, “limited-stage”, “radiotherapy (or irradiation)”, and “chemotherapy”, with no constraints imposed on the timeframe for the search, for randomised evidence to support this practice. We found only one relevant randomised clinical trial comparing once-daily and twice-daily radiotherapy.

**Added value of this study**

Although twice-daily radiotherapy has produced the best outcomes in these patients so far, concerns about its toxicity, logistical issues in the delivery of twice-daily radiotherapy, and the low radiation dose used in the control group of the Intergroup 0096 study have resulted in the poor adoption of this regimen and no consensus on the standard treatment to use in the routine setting. The CONVERT trial provides further evidence supporting the use of twice-daily radiotherapy in the routine setting and will help to standardise patient care. Furthermore, the results of this study show that in the era of modern radiotherapy techniques, the frequency and severity of acute and late radiation toxicities are lower than previously reported.

**Implications of all the available evidence**

Results from this study showed that twice-daily radiotherapy should be considered standard-of-care in patients with limited-stage small-cell lung cancer. The implication for future research is that overall treatment duration of radiotherapy should be kept short when combined with chemotherapy. This Article provides updated information on expected treatment toxicity that clinicians can relay to their patients.

## Introduction

Small-cell lung cancer is characterised by its rapid tumour doubling time, early dissemination, and high response rate to both chemotherapy and radiotherapy. Of the 42 000 patients in the UK and 225 000 in the USA diagnosed with lung cancer every year, 15% have small-cell lung cancer and 30% of those have limited-stage disease that can be encompassed within a tolerable radiotherapy field.^1^ Even in this early-stage disease, outcomes are poor, with median survival of 16–24 months after curative intent treatment and 2-year survival of less than 50%.^2^, ^3^, ^4^ Combined chemotherapy and thoracic radiotherapy is the standard treatment for limited-stage small-cell lung cancer. Results from two meta-analyses^5^, ^6^ showed that the addition of radiotherapy to chemotherapy improves median survival, 3-year survival, and local control. Subsequently, meta-analyses of clinical trials investigating the optimal timing and sequencing of chemoradiotherapy have shown an advantage for early concurrent thoracic radiotherapy.^7^, ^8^, ^9^, ^10^, ^11^ Furthermore, twice-daily radiotherapy was superior to once-daily radiotherapy in the landmark Intergroup 0096 study.^4^ In that study, patients were randomly assigned to receive either 45 Gy once-daily (1·8 Gy per fraction) for 5 weeks or 45 Gy twice-daily (1·5 Gy per fraction) for 3 weeks. In both groups, radiotherapy was given concurrently, starting with the first cycle of chemotherapy. Twice-daily radiotherapy significantly improved 5-year overall survival compared with once-daily radiotherapy (26% *vs* 16%) and reduced the risk of thoracic relapse (36% *vs* 52%) but at the cost of increased severe radiation oesophagitis (32% *vs* 16%). Consequently, twice-daily radiotherapy concurrently with chemotherapy was adopted as a standard of care for limited-stage small-cell lung cancer.^12^ However, it is unclear whether twice-daily radiotherapy resulted in better outcomes because of the increase in the biologically effective dose of radiation or because of shorter overall treatment time, which is important in this rapidly proliferating disease. Radiotherapy techniques have evolved since the Intergroup 0096 study was designed in the late 1980s; specifically, the use of CT-planned conformal treatment and the omission of elective nodal irradiation to reduce normal tissue exposure and toxicity, particularly oesophagitis.

Although twice-daily radiotherapy concurrently with chemotherapy has produced the best outcomes so far, concerns about its toxicity, logistical issues in its delivery, and the low radiation dose in the control group of the Intergroup 0096 study, resulting in a very high (52%) local failure rate, have resulted in the poor adoption of this regimen and no consensus on the standard treatment to use in the routine setting.^13^ The authors of one study^14^ suggested that the local control could be improved with a higher dose of once-daily radiotherapy. The CONVERT trial was therefore designed as a superiority trial to improve on the standard of care for limited-stage small-cell lung cancer by comparing twice-daily radiotherapy to a higher dose of radiotherapy delivered once daily, given concurrently with chemotherapy.

## Methods

### Study design and participants

The CONVERT trial was an international, multicentre, open-label, randomised phase 3 superiority trial. Details of the trial design have been published previously.^15^ Patients were recruited at 73 centres in eight countries (Belgium, Canada, France, Netherlands, Poland, Slovenia, Spain, and the UK; appendix pp 1–2).

Eligible patients were aged 18 years or older; had histologically or cytologically confirmed small-cell lung cancer with limited disease (as defined by the Veterans Administration Lung Cancer Study Group—ie, patients whose disease can be encompassed within a radical radiation portal);^16^ had an Eastern Cooperative Oncology Group performance status of 0–1^17^ or performance status of 2 due to disease-related symptoms and not comorbidities (since small-cell lung cancer is characterised by rapid doubling time and central disease location, which can be associated with a sudden change in performance status); had no malignant pleural or pericardial effusions; and had acceptable radiotherapy target volume (according to the local radiotherapist). Eligible patients had a maximum of one adverse biochemical factor (concentrations of serum alkaline phosphatase >1·5-times the upper limit of normal, serum sodium <lower limit of normal, and serum lactate dehydrogenase >the upper limit of normal), forced expiratory volume in 1 s greater than 1 L or 40% predicted value, and transfer factor for carbon monoxide greater than 40% predicted value. Patients with a previous history of malignancy in the past 5 years (except for non-melanomatous skin or in-situ cervix carcinoma) and those with previous or concomitant illness or treatment that, in the opinion of the investigator, would interfere with the trial treatments or comparisons were excluded.

Participants gave written informed consent and the study was done according to the Declaration of Helsinki and Good Clinical Practice Guidelines. The trial was reviewed in the UK by the National Research Ethics Service Committee North West–Greater Manchester Central, which granted ethics approval for the study on Dec 21, 2007 (REC reference: 07/H1008/229). The protocol was also approved by the institutional review board or research ethics committee in each country and at each study centre.

### Randomisation and masking

Patients were randomly assigned (1:1) to one of the two treatment groups (twice-daily *vs* once-daily radiotherapy). Allocation to treatment group was done by phone call or fax from the recruiting centre to the Manchester Academic Health Science Centre Trials Coordination Unit. The allocation method used was minimisation with a random element using a bespoke computer application. The factors controlled for in the allocation were institution, planned number of chemotherapy cycles (four *vs* six), and performance status (0–1 *vs* 2). Patients and investigators were not masked to treatment allocation.

### Procedures

At baseline, all patients underwent baseline investigations, which included physical examination, chest radiograph, CT scan of the thorax and upper abdomen, CT or MRI of the brain, full blood count, biochemical profile, and lung function tests. PET/CT scans were allowed but not mandatory. Staging was done using the Union for International Cancer Control/American Joint Committee on Cancer classification system.^18^

Patients were randomly assigned to receive radiotherapy either twice-daily (45 Gy in 30 twice-daily fractions of 1·5 Gy, with a minimum of 6 h between fractions, over 19 days, on 5 consecutive days a week) or once-daily (66 Gy in 33 daily fractions of 2 Gy over 45 days, on 5 consecutive days a week), concurrently with chemotherapy. Chemotherapy was started within 4 weeks of randomisation and consisted of four to six cycles of cisplatin and etoposide every 3 weeks in both groups (etoposide 100 mg/m^2^ intravenously on days 1–3 and cisplatin 75 mg/m^2^ intravenously on day 1, or etoposide 100 mg/m^2^ intravenously on days 1–3 and cisplatin 25 mg/m^2^ intravenously on days 1–3). Each centre had to elect to prescribe four or six cycles for all eligible trial patients. The first cycle of chemotherapy was given before radiotherapy and the second was given concurrently with radiotherapy if no delay with chemotherapy occurred. No later than 6 weeks after the last cycle of chemotherapy, patients without evidence of progressive disease on the CT scan and with no clinical evidence of brain metastases were offered prophylactic cranial irradiation.

Radiotherapy commenced on day 22 of cycle one of chemotherapy, coinciding with cycle two of chemotherapy in patients not experiencing chemotherapy delay due to toxicity. 3D conformal radiotherapy was mandatory and elective nodal irradiation was not permitted. The total dose was prescribed at the International Commission on Radiation Units and Measurements reference point. Intensity-modulated radiotherapy and PET/CT planning was permitted but not mandated. The protocol specified that if dose constraints to the organs at risk could not be met, the dose delivered could be decreased accordingly. The policy for chemotherapy was to delay and give at full dose later, rather than give at a reduced dose. However, we recommended a chemotherapy treatment delay of more than 7 days for grade 4 febrile neutropenia, grade 4 thrombocytopenia requiring medical intervention, or grade 2 or worse bleeding with thrombocytopenia; for the first episode of such an event, we recommended full-dose chemotherapy and granulocyte colony-stimulating factor support, or a 20% dose reduction. In case of a second episode, we recommended a 30% dose reduction. If a third episode occurred, the patient was removed from the trial.

A radiotherapy quality assurance programme was set up to ensure the robustness of the radiotherapy procedures, and the details of the programme have been reported previously.^15^ The programme was managed by the UK National Cancer Research Institute Radiotherapy Trials Quality Assurance Team.

On completion of study treatment, patients were followed up weekly until the resolution of acute side-effects, then every 3 months until 1 year, and every 6 months for 5 years. A CT scan of the thorax and abdomen was done at 4 weeks after cycle four (even if six cycles were given). Subsequently, during follow-up at 6 and 12 months after randomisation, investigations included physical evaluation, reporting of adverse events, and a CT scan of the thorax and abdomen. Follow-up investigations were done according to local policy.

### Outcomes

The primary outcome of the study was overall survival, defined as time from randomisation until death from any cause. Secondary outcomes included compliance with chemotherapy and radiotherapy (defined as dose intensity delivered), acute toxicity (defined as toxicity occurring between the start of treatment and up to 3 months after completion of treatment, and assessed according to the Common Terminology Criteria for Adverse Events [version 3.0]), late toxicity (according to the Common Terminology Criteria for Adverse Events [version 3.0]),^19^ and local and metastatic progression-free survival (calculated from date of randomisation to date of first clinical or radiological evidence of progressive disease at the primary site or distant sites). With regard to toxicity, frequencies of worst recorded grade of toxicity in the respective time periods were recorded. Response rate was another secondary outcome but it was not analysed because interpretation of CT imaging would have been too complex after concurrent chemoradiotherapy. The study also had post-hoc exploratory translational objectives, which will be reported at a later date. All serious adverse events were reported to the trial coordinating centre and were assessed for causality and expectedness, both locally by the Principal Investigator and centrally by the Chief Investigator.

### Statistical analysis

Our hypothesis was that overall survival in the once-daily chemoradiotherapy group would be superior to that of the twice-daily group. A 12% higher overall survival at 2 years in the once-daily group versus the twice-daily group was considered to be clinically significant to show superiority of the once-daily regimen. Overall and progression-free survival were estimated with the Kaplan-Meier method, and between-group comparisons evaluated by the log-rank test with stratification for institution, planned number of chemotherapy cycles (four *vs* six), and performance status (0–1 *vs* 2). The number of events required to detect a hazard ratio (HR) for death of 0·7 with an α level (two-sided) of 0·05 and 80% power (ie, an increase in 2-year survival from 44% in the twice-daily radiotherapy group to 56% in the once-daily radiotherapy group) was 247. An additional 5% was added to the sample size of 506 patients to allow for ineligible patients, giving a total recruitment target of 532 patients. The primary survival outcome was analysed using the modified intention-to-treat principle, because four cases provided no follow-up data and hence were censored at time zero. Further details about the statistical analysis are available in the protocol.^15^ All randomly assigned patients who were treated with at least one study dose of chemotherapy and who were alive at the time of the first toxicity assessment were included in the safety analysis. Data were collected at each study site and monitored by the independent data monitoring committee. We submitted reports to the independent data monitoring committee on an annual basis, commencing 12 months after the first patient was randomly assigned. The statistical package used for the analyses was Stata (version 13.1).

This trial is registered with ISRCTN, number 91927162, and ClinicalTrials.gov, number NCT00433563.

### Role of the funding source

Cancer Research UK reviewed and approved the study design. None of the funders had a role in data collection, data analysis, data interpretation, or writing of the report. The corresponding author had full access to all the data in the study and had final responsibility for the decision to submit for publication.

## Results

**Figure 1:**
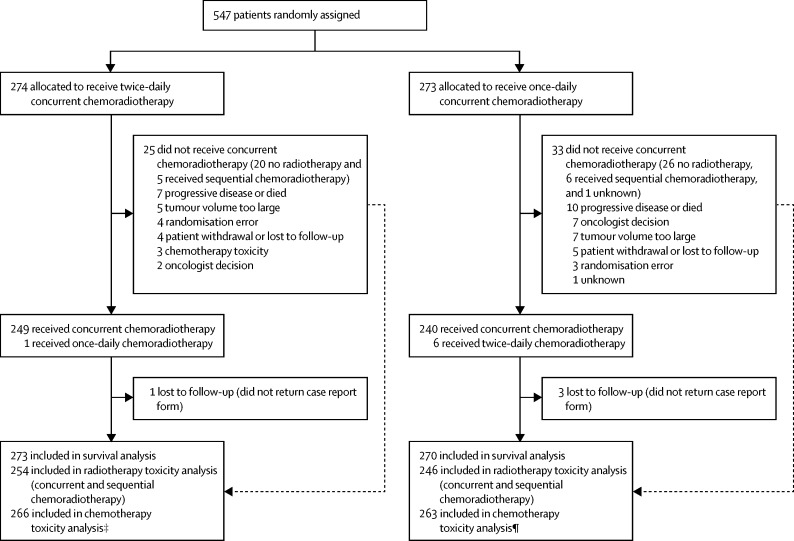
Trial profile *One patient withdrew consent for twice-daily radiotherapy. †Dose constraints to organs at risk not met in four patients and twice-daily radiotherapy given in error to two patients. ‡Six patients did not receive any chemotherapy and two patients died during cycle one before toxicity assessment. ¶Seven patients did not receive any chemotherapy and three patients died during cycle one before toxicity assessment. Numbers assessed and ineligible are unavailable because screening logs were not completed by all centres.

Between April 7, 2008, and Nov 29, 2013, we recruited 547 patients from 73 centres in eight countries. We randomly assigned 274 patients to receive twice-daily chemoradiotherapy and 273 to receive once-daily chemoradiotherapy. The modified intention-to-treat survival analysis included 543 patients (273 in the twice-daily chemoradiotherapy group and 270 in the once-daily chemoradiotherapy group) because four patients were lost to follow-up (centres did not return their case report forms; (figure 1). Table 1 shows the baseline characteristics of the participants. The median age at randomisation was 62 years (IQR 29–84) in the twice-daily group and 63 years (34–81) in the once-daily group, with 83 (15%) of 547 patients being older than 70 years (32 [12%] in the twice-daily group and 51 [19%] in the once-daily group). More than 95% of patients overall had a performance status of 0–1. Less than 2% of patients were never smokers, almost two-thirds were former smokers, and just over a third were current smokers (table 1). 312 (57%) of 547 patients were staged with PET/CT, and 426 (78%) of 547 were stage III according to the Union for International Cancer Control classification (table 1).

**Table 1:** Baseline characteristics

		**Twice-daily radiotherapy (n=274)**	**Once-daily radiotherapy (n=273)**
Age (years)		62 (29–84)	63 (34–81)
Sex			
	Male	147 (54%)	150 (55%)
	Female	127 (46%)	123 (45%)
Ethnicity			
	White	262 (96%)	265 (97%)
	African	1 (<1%)	1 (<1%)
	Asian	1 (<1%)	4 (2%)
	Other	6 (2%)	3 (1%)
	Not known	4 (2%)	0
Eastern Cooperative Oncology Group performance status			
	0	125 (46%)	123 (45%)
	1	137 (50%)	142 (52%)
	2	9 (3%)	8 (3%)
	Not known*	3 (1%)	0
Smoking history†			
	Never smoker	3 (1%)	4 (2%)
	Former smoker	174 (64%)	163 (60%)
	Current smoker	94 (34%)	106 (39%)
	Not known	3 (1%)	0
Adverse biochemical factors			
	Elevated lactate dehydrogenase	69 (25%)	60 (22%)
	Hyponatraemia	57 (21%)	53 (19%)
	Elevated alkaline phosphate	5 (2%)	6 (2%)
PET/CT staging			
	Yes	157 (57%)	155 (57%)
	No	113 (41%)	118 (43%)
	Not known	4 (2%)	0
UICC/AJCC stage^18^			
	I	1 (<1%)	3 (1%)
	II	34 (12%)	48 (18%)
	III	219 (80%)	207 (76%)
	Not known	20 (7%)	15 (6%)
Gross tumour volume (cc)		81·6 (1·6–635·1)	85·6 (0·5–593·0)
Number of chemotherapy cycles planned			
	Four	188 (69%)	183 (67%)
	Six	86 (31%)	90 (33%)

Data are median (IQR) or n (%). UICC/AJCC=Union for International Cancer Control/American Joint Committee on Cancer.

* Eastern Cooperative Oncology Group Performance Status was not recorded on the source documentation and case report form in three cases at baseline; in all three cases, the performance score was recorded as 0–1 on the randomisation form.

† Never smokers defined as adults who have never smoked a cigarette or who smoked fewer than 100 cigarettes in their entire lifetime; former smokers defined as adults who have smoked at least 100 cigarettes in their lifetime but say they currently do not smoke; current smokers defined as adults who have smoked 100 cigarettes in their lifetime and currently smoke cigarettes every day (daily) or on some days (non-daily).

The number of planned cycles of chemotherapy was four for most patients (table 1). Almost 60% of patients actually received four cycles and a further 20% received six cycles of chemotherapy (table 2).

**Table 2:** Treatment delivered

		**Twice-daily radiotherapy (n=274)**	**Once-daily radiotherapy (n=273)**	p value*
Chemotherapy cycles delivered (all patients)				0·89
	0	6 (2%)	7 (3%)	
	1	15 (6%)	15 (6%)	
	2	8 (3%)	6 (2%)	
	3	23 (8%)	24 (9%)	
	4	161 (59%)	156 (57%)	
	5	5 (2%)	12 (4%)	
	6	56 (20%)	53 (19%)	
Radiotherapy treatment				0·60
	Concurrent chemoradiotherapy	249 (91%)	240 (88%)	
	Sequential chemoradiotherapy	5 (2%)	6 (2%)	
	No radiotherapy	20 (7%)	26 (10%)	
	Not known	**..**	1 (<1%)	
Chemotherapy cycles delivered in patients who received concurrent chemoradiotherapy†				
	1	3/249 (1%)	1/240 (<1%)	
	2	5/249 (2%)	5/240 (2%)	
	3	21/249 (8%)	20/240 (8%)	
	4	161/249 (66%)	150/240 (63%)	
	5	5/249 (2%)	12/240 (5%)	
	6	54/249 (21%)	52/240 (22%)	
Intensity-modulated radiotherapy				0·59
	Yes	40/254‡ (16%)	43/247‡ (17%)	
	Not known	**..**	1 (<1%)	
Prophylactic cranial irradiation		229 (84%)	220 (81%)	0·36

Data are n (%) or n/N (%).

* All p values were calculated with χ^2^ tests (except for number of cycles, which is a Wilcoxon rank sum test).

† The denominator in each group is the number of patients who received concurrent chemoradiotherapy.

‡ The denominator in each group is the number of patients who received radiotherapy.

At the data analysis cutoff in March 1, 2016, the median follow-up was 45 months (IQR 35–58) for those still alive. 164 (60%) of 273 patients in the twice-daily group had died, compared with 176 (65%) of 270 patients in the once-daily group.

**Figure 2:**
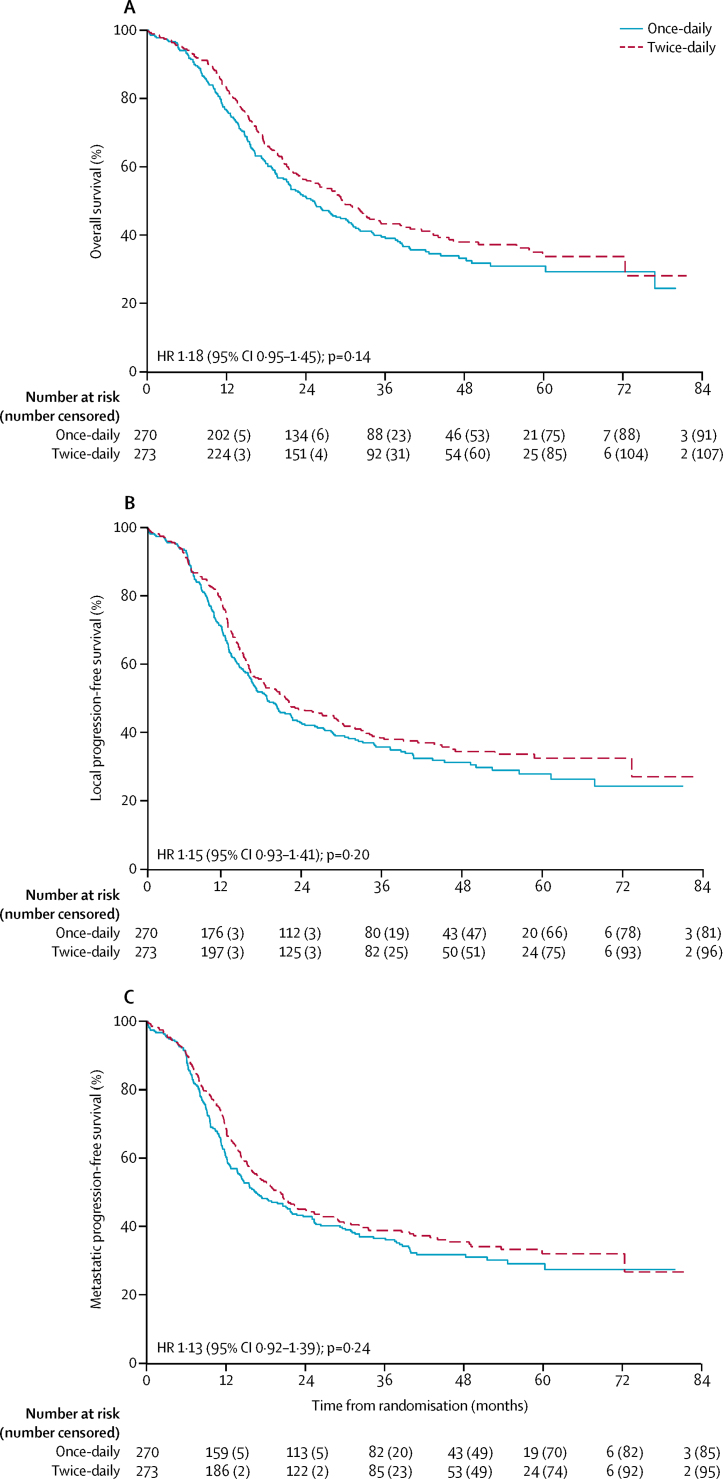
Overall and progression-free survival (A) Overall survival. (B) Local progression-free survival. (C) Metastatic progression-free survival. HR=hazard ratio.

In our survival analysis (which included 273 patients in the twice-daily group and 270 in the once-daily group), median overall survival was 30 months (95% CI 24–34) in the twice-daily group and 25 months (21–31) in the once-daily group (hazard ratio 1·18 [95% CI 0·95–1·45]; p=0·14; figure 2A). 2-year overall survival was 56% (95% CI 50–62) in the twice-daily group and 51% (45–57) in the once-daily group (absolute difference between the treatment groups 5·3% [95% CI −3·2% to 13·7%]). 5-year overall survival was 34% (95% CI 27–41) in the twice-daily group and 31% (25–37) in the once-daily group (absolute difference 2·8% [95% CI −6·4% to 12·0%]). In the twice-daily group versus the once-daily group, causes of death were lung cancer (152 *vs* 146), intercurrent deaths (six *vs* 14), treatment-related (three *vs* eight), and cardiovascular (three *vs* eight); causes of the 12 treatment-related deaths were radiation pneumonitis (one *vs* two), dementia possibly related to prophylactic cranial irradiation (none *vs* one), neutropenic sepsis (one *vs* three), septic shock (one *vs* none), bronchial pneumonia (none *vs* two), and peripheral vascalar ischaemia (one *vs* none).

25 (9%) of 273 patients in the twice-daily radiotherapy group and 33 (12%) of 270 in the once-daily radiotherapy group did not receive concurrent chemoradiotherapy (figure 1), giving compliance rates of 91% in the twice-daily group and 88% in the once-daily group. Less than 10% of patients did not receive any radiotherapy (20 [7%] in the twice-daily group and 26 [10%] in the once-daily group; figure 1, table 2). Of the patients who received radiotherapy, intensity-modulated radiotherapy was delivered to 40 (16%) of 254 participants in the twice-daily group versus 43 (17%) of 247 participants in the once-daily group. Prophylactic cranial irradiation was delivered to 229 (84%) of 274 *vs* 220 (81%) of 273 participants (table 2). More patients received the full dose of radiotherapy in the twice-daily group than in the once-daily group (p<0·0001; table 3). The optimal number of fractions, as defined in the protocol,^15^ (30 fractions in the twice daily group and 33 in the once daily group) were delivered in 213 (86%) of 249 patients in the twice-daily group and 192 (80%) of 240 patients in the once-daily group (p=0·10). Radiotherapy was delivered over the planned overall treatment time of 19 days in 158 (63%) of 249 patients in the twice-daily group and over the planned overall treatment time of 45 days in 114 (48%) of 240 patients in the once-daily group (p=0·0004). Protocol deviations and violations were mainly due to logistical reasons, such as public holidays.

**Table 3:** Radiotherapy treatment delivered in patients receiving concurrent chemoradiotherapy (as per protocol)

	**Dose (Gy)**						**Number of fractions**								**Overall treatment time (days)**							
	<44	44–46*	>46	<60	60–62	64–68*	<28	28–29	30†	>30	<30	30–32	33†	>33	<19	19‡	20–21§	>21¶	<45	45‡	46–47§	>47¶
Twice-daily radiotherapy (n=249)	1 (<1%)	245 (98%)	3 (1%)	**..**	**..**	**..**	12 (5%)	23 (9%)	213 (86%)	1 (<1%)	**..**	**..**	**..**	**..**	15 (6%)	158 (63%)	24 (10%)	52 (20%)	**..**	**..**	**..**	**..**
Once-daily radiotherapy (n=240)	**..**	**..**	**..**	22 (9%)	19 (8%)	199 (83%)	**..**	**..**	**..**	**..**	16 (7%)	31 (13%)	192 (80%)	1 (<1%)	**..**	**..**	**..**	**..**	41 (17%)	114 (48%)	43 (18%)	42 (18%)

Data are n (%).

* Full dose.

† Optimal number of fractions, as defined in the protocol.

‡ Planned overall treatment time.

§ Deviation.

¶ Violation.

At the time of analysis, 181 (66%) of 273 patients in the twice-daily group and 189 (70%) of 270 patients in the once-daily group had disease progression (p=0·26). Median progression-free survival was 15·4 months (95% CI 13·7–19·8) in the twice-daily group and 14·3 months (12·0–17·0) in the once-daily group (hazard ratio 1·12 [95% CI 0·92–1·38]; p=0·26). Median local progression-free survival was 20·7 months (95% CI 16·1–27·9) in the twice-daily group versus 17·9 months (15·3–21·7) in the once daily group (figure 2B) and median metastatic progression-free survival was 20·2 months (95% CI 15·9–25·3) versus 16·6 months (13·7–21·8). The difference between groups for local progression-free survival (p=0·20) and metastatic progression-free survival (p=0·24) was not significant (figure 2). There was no notable difference between groups in treatment received at the time of progression (appendix p 4).

Chemotherapy toxicity was assessed in 266 (97%) of 273 patients in the twice-daily group and 263 (97%) of 270 patients in the once-daily group, who had received at least one cycle of chemotherapy and who were alive at the time of the first toxicity assessment (figure 1, table 4). Radiotherapy toxicity was assessed in 254 (93%) of 273 patients in the twice-daily group and 246 (91%) of 270 patients in the once-daily group who had received either concurrent or sequential chemoradiotherapy (figure 1, table 4).

**Table 4:** Acute adverse events (≤3 months after completion of study treatment)

	**Twice-daily group**				**Once-daily group**				**p value**
	Grade 1–2	Grade 3	Grade 4	Grade 5	Grade 1–2	Grade 3	Grade 4	Grade 5	
**Adverse events in the population assessed for chemotherapy toxicity (n=266 in the twice-daily group; n=263 in the once-daily group)**									
Nausea	172 (65%)	23 (9%)	..	..	171 (65%)	26 (10%)	..	..	0·63
Vomiting	105 (40%)	13 (5%)	..	..	95 (36%)	13 (5%)	..	..	0·99
Mucositis	88 (33%)	3 (1%)	..	..	87 (33%)	5 (2%)	1 (<1%)	..	0·34
Fatigue	212 (80%)	31 (12%)	..	..	216 (82%)	31 (12%)	2 (1%)	..	0·77
Neuropathy (motor)	12 (5%)	1 (<1%)	..	..	15 (6%)	2 (1%)	..	..	0·62
Neuropathy (sensory)	63 (24%)	3 (1%)	1 (<1%)	1 (<1%)	61 (23%)	5 (2%)	..	..	>0·99
Infection	43 (16%)	27 (10%)	7 (3%)	..	52 (20%)	27 (10%)	2 (1%)	2 (1%)	0·52
Anaemia	194 (73%)	32 (12%)	1 (<1%)	..	184 (70%)	34 (13%)	1 (<1%)	..	0·72
Febrile neutropenia	NA	49 (18%)	13 (5%)	1 (<1%)	NA	38 (14%)	8 (3%)	3 (<1%)	0·13
Neutropenia	38 (14%)	68 (26%)	129 (49%)	..	47 (18%)	69 (26%)	101 (38%)	..	0·05
Anorexia	135 (51%)	18 (7%)	..	..	129 (49%)	21 (8%)	..	..	0·60
Other*	150 (57%)	65 (24%)	9 (3%)	1 (<1%)†	177 (67%)	44 (17%)	8 (3%)‡	1 (<1%)	0·02
**Adverse events in the population assessed for radiotherapy toxicity (n=254 in the twice-daily group; n=246 in the once-daily group)**									
Oesophagitis	159 (63%)	46 (18%)	1 (<1%)	..	135 (54%)	47 (19%)	..	..	0·85
Pneumonitis	51 (20%)	3 (1%)	1 (<1%)	1 (<1%)	49 (19%)	3 (1%)	1 (<1%)	2 (1%)	0·70

Data are n (%). The radiotherapy toxicity population was used to analyse the prevalence of these adverse events because it would not be possible to report radiotherapy-related toxicity in patients who did not receive radiotherapy. NA=not applicable.

* Other grade 3 reported toxicities included diarrhoea (n=7), hyponatremia (n=1), urinary retention (n=5), dysphagia (n=5), and lymphopenia (n=6) in the once-daily group; and diarrhoea (n=3), constipation (n=7), hyponatremia (n=1), dysphagia (n=8), lymphopenia (n=8), dyspnoea (n=8), and leucopenia (n=4) in the twice-daily group. Other grade 4 reported toxicities included pulmonary embolism (n=4), hyponatremia (n=2), dyspnoea (n=1), and myocardial infarction (n=1) in the once-daily group; and pulmonary embolism (n=2), hyponatremia (n=3), lymphopenia (n=3), and fast atrial fibrillation (n=1) in the once-daily group).

† Two deaths (peripheral arterial ischaemia [n=1] and septic shock [n=1]).

‡ Two deaths (peripheral arterial ischaemia [n=1] in the twice-daily group and dementia possibly related to prophylactic cranial irradiation [n=1] in the once-daily group).

The most common grade 3–4 adverse event was neutropenia (affecting 197 [74%] of 266 patients in the twice-daily group *vs* 170 [65%] of 263 in the once-daily group). The frequencies of most adverse events recorded were similar in both groups, with the exception that significantly more grade 4 neutropenia was recorded in the twice-daily group than in the once-daily group (129 [49%] *vs* 101 [38%]; p=0·05). However, grade 3–5 febrile neutropenia did not differ significantly between the two groups (table 4). Acute radiotherapy toxicity was similar in both groups: grade 3–4 oesophagitis was reported in 47 (18%) of 254 patients in the twice-daily group and 47 (19%) of 246 patients in the once-daily group. 11 patients developed grade 3–5 radiation pneumonitis (five in the twice daily group and six in the once daily group), of whom three patients died within 3 months of radiotherapy (two in the once-daily group and one in the twice-daily group, one of whom received sequential rather than concurrent radiotherapy; table 4, appendix p 3). Regarding late toxicity, four patients in the once-daily group developed grade 3 oesophagitis, one of whom had an oesophageal stricture. Six patients in each group developed grade 3–4 pneumonitis, and five patients (three in the twice-daily group and two in the once-daily group) developed grade 3 pulmonary fibrosis (table 5).

**Table 5:** Late adverse events (>3 months after study treatment)

	**Twice-daily group (n=248)**			**Once-daily group (n=233)**			p value*
	Grade 1–2	Grade 3	Grade 4	Grade 1–2	Grade 3	Grade 4	
Dermatitis	15 (6%)	..	..	17 (7%)	..	..	..
Oesophagitis	29 (12%)	..	..	39 (17%)	4 (2%)	..	0·06
Oesophageal stricture or fistula	8 (3%)	..	..	6 (3%)	1 (<1%)	..	0·48
Pulmonary fibrosis	119 (48%)	3 (1%)	..	106 (46%)	2 (1%)	..	>0·99
Pneumonitis	71 (29%)	5 (2%)	1 (<1%)	70 (30%)	5 (2%)	1 (<1%)	0·90
Myelitis	1 (<1%)†	..	..	8 (3%)†	..	..	..
Other	131 (53%)	20 (8%)	3 (1%)	113 (49%)	18 (8%)	2 (1%)	0·78

Data are n (%).

* p values calculated for grade 3–4 adverse events.

† All cases of myelitis were grade 1 adverse events.

## Discussion

Our results show that once-daily radiotherapy did not improve overall survival in patients with limited-stage small-cell lung cancer and good performance status, compared with twice-daily radiotherapy, when given concurrently with chemotherapy. Radiotherapy treatment delivery was superior in the twice-daily group. Furthermore, both acute and late toxicities were similar and lower than expected with both regimens.

However, although results are unable to show superiority of the once-daily radiotherapy regimen, the CONVERT trial should have a major effect on the standardisation of chemoradiotherapy in this disease group—a treatment that has been the subject of controversy since the publication of the Intergroup 0096 study.^4^, ^13^ Overall survival with both regimens were higher than the survival results reported in the Intergroup 0096 study. In CONVERT, 2-year survival for twice-daily and once-daily radiotherapy was 56% and 51%, versus 47% and 41% in the Intergroup 0096 study.^4^ CONVERT was not an equivalence study (and was not powered for equivalence) so it cannot be concluded that the two regimens have the same efficacy. Furthermore, the 2-year survival of 56% achieved in the control group with twice-daily radiotherapy is the same survival that was projected for the experimental group. The better-than-expected performance of both groups might be explained by several changes in the management of small-cell lung cancer since the publication of the Intergroup study, including PET/CT staging in more than half of patients, the use of modern and precise radiotherapy techniques, and improvements in supportive care. These results, together with several meta-analyses and systematic overviews, support the use of short overall radiotherapy treatment time to avoid early cancer cell repopulation.^7^, ^8^, ^9^, ^10^, ^11^ One of the systematic overviews also identified that time from the start of any treatment to completion of radiotherapy is a key variable in predicting outcome.^20^ Although not significant, 2-year overall survival was slightly higher in the twice-daily group than in the once-daily group, which could possibly be a result of improved delivery of treatment in the twice-daily group, with more patients receiving full-dose radiotherapy, the optimal planned number of fractions, and treatment delivered over the optimal treatment time. Another reason why treatment delivery was superior in the twice-daily group is because of the lower overall dose of radiotherapy in this group, which meant it was possible to achieve the protocol dose constraints for organs at risk, such as lung and spinal cord, in a greater proportion of patients than in the once-daily group. A further advantage of the twice-daily regime is that it halves the radiotherapy treatment time (from 45 days to 19 days) and reduces the number of fractions (from 33 to 30) compared with the once-daily regimen. Although no formal health economic analysis has been done as part of this study, the delivery of twice-daily radiotherapy could lead to cost savings, especially if patients require hospital transport to attend radiotherapy appointments.

Overall, the frequency and severity of acute and late radiation toxicities were lower than expected, probably because of the use of modern radiotherapy techniques, including 3D radiotherapy or intensity-modulated radiotherapy, and treatment of involved fields with regard to nodal disease. In the Intergroup 0096 trial,^4^ patients were treated with outdated radiotherapy techniques including elective nodal irradiation, which would have resulted in higher radiation exposure of normal tissues than in this trial. Indeed, the high rate of severe acute oesophagitis (32% with twice-daily radiotherapy) in the Intergroup study has been cited as the main reason for poor adoption of twice-daily radiotherapy.^13^ By contrast, less than 20% of patients had severe oesophagitis in the CONVERT study and only one patient developed an oesophageal stricture requiring intervention. Radiation pneumonitis was not specifically reported in the Intergroup 0096 study, but in this trial very few (<3%) patients had severe radiation pneumonitis or severe pulmonary fibrosis. The lower than anticipated toxicity rates and rates of local failure reported in this study suggest that radiotherapy treatment delivered concurrently with chemotherapy could be intensified further—for example, by means of dose escalation or hypofractionation.

A limitation of this study is that although we did not mandate an upper age limit—with the aim to gather much needed evidence about the outcome of elderly patients treated with concurrent chemoradiotherapy—only 15% of the patients included were older than 70 years. Data for patients older than 70 years participating in CONVERT was presented at the International Association for the Study of Lung Cancer 17th World Conference on Lung Cancer in Vienna, Austria, in 2016, and the results of this analysis will be presented in a separate report. Elderly patients have been reported to be less likely to receive concurrent chemoradiotherapy than their younger counterparts, which is mainly due to insufficient high-quality evidence to support the use of this potentially toxic treatment.^21^ Another limitation is that the majority of patients enrolled in both groups were white, and therefore the results of the study might not be applicable to other ethnicities.

To our knowledge, CONVERT is the largest study completed investigating thoracic radiotherapy in limited-stage small-cell lung cancer, and the first clinical trial in this group of patients to report on the outcome of patients treated with modern radiotherapy techniques incorporating a quality assurance programme. It was possible to complete this study because of the interest, enthusiasm, and collaborative efforts of a large number of investigators from many different countries. The key to completing accrual was to include a large number of recruiting sites. Furthermore, by contrast with US practice, concurrent chemoradiotherapy is not always adopted as the standard of care for limited-stage small-cell lung cancer in Europe, and the study provided an incentive for centres to adopt and set up concurrent treatment protocols.

Given the importance of keeping overall treatment time as short as possible, future studies could investigate dose-escalated twice-daily or hypofractionated radiotherapy concurrently with chemotherapy. Further data for the outcome of patients treated with high-dose 2 Gy per fraction treatment will be provided by the ongoing CALGB 30610/RTOG 0538 study (NCT00632853). The upcoming analysis of the CONVERT translational studies, including the prognostic role of baseline circulating tumour cells, could provide data for relevant biological stratification factors that can be used prospectively in future studies.

In conclusion, the results of CONVERT show that there were no significant differences in survival and no major differences in toxicity between twice-daily and once-daily radiotherapy. However, since the trial was designed to show superiority of once-daily radiotherapy and not powered to show equivalence, twice-daily radiotherapy should continue to be considered standard-of-care. Furthermore, twice-daily radiotherapy concurrently with chemotherapy is well tolerated, with better compliance and shorter treatment time than once-daily treatment. From a pragmatic perspective, once-daily radiotherapy could be considered when delivery of twice-daily radiotherapy is impossible because of departmental logistics or patient choice.
