# Supplementary material for: Concurrent once-daily versus twice-daily chemoradiotherapy in patients with limited-stage small-cell lung cancer (CONVERT): an open-label, phase 3, randomised, superiority trial
Source: Lancet Oncol. 2017 Aug;18(8):1116–25. doi: 10.1016/S1470-2045(17)30318-2 (PMC5555437; doi:10.1016/S1470-2045(17)30318-2)
Supplement: Supplementary appendix [file mmc1.pdf]

THE LANCET
Oncology

Supplementary appendix

This appendix formed part of the original submission and has been peer reviewed. We post it as supplied by the authors.

Supplement to: Faivre-Finn C, Snee M, Ashcroft L, et al, for the CONVERT Study Team. Concurrent once-daily versus twice-daily chemoradiotherapy in patients with limited-stage small-cell lung cancer (CONVERT): an open-label, phase 3, randomised, superiority trial. *Lancet Public Health* 2017; published online June 19. http://dx.doi.org/10.1016/S1470-2045(17)30318-2.

Web appendix

Details of Recruiting Centres, Numbers of Patients Recruited (N) and Principal Investigators.

UK – 33 Sites

- The Christie NHS Foundation Trust, Manchester (109) – Faivre-Finn
- Edinburgh Cancer Centre (6) – Price
- Bristol Haematology & Oncology Centre (10) – Wilson
- Dorset Cancer Centre, Poole (5) – Laurence
- Torbay Hospital, Devon (2) – Dorey
- Weston Park Hospital, Sheffield (14) – Woll/Hatton
- Lancashire Teaching Hospitals NHS Foundation Trust (15) – Appel
- Cheltenham General Hospital (3) - Guglani
- St James Hospital, Leeds (18) – Snee
- Hammersmith Hospital, London (1) – Mangar
- University College London Hospitals (3) – Carnell
- Beatson West of Scotland Cancer Centre, Glasgow (19) – Mohammed
- Glan Clwyd Hospital, North Wales (10) - Garcia
- Southampton General Hospital (14) – Bhatnagar
- Castle Hill Hospital, Hull (3) – Lind
- James Cook Hospital, Middlesbrough (3) – Peedell
- Northern Ireland Cancer Centre, Belfast City Hospital (10) – McAleese
- Royal Marsden Hospital, Surrey (18) – O'Brien/Locke
- Norfolk & Norwich University Hospital (1) – Roques
- Addenbrooke's Hospital, Cambridge (15) – Harden
- St Bartholomew's Hospital, London (3) - Wells
- Raigmore Hospital, Inverness (1) – Macgregor
- Bradford Royal Infirmary (4) – Snee
- Queen Elizabeth Hospital, Birmingham (2) – Chetiyawardana
- Peterborough City Hospital (2) – Fife
- Northern Centre for Cancer Care, Newcastle-upon-Tyne (20) – McMenemin
- Harrogate District NHS Foundation Trust (1) – Chan
- Derriford Hospital, Plymouth (1) – Roy
- The Clatterbridge Cancer Centre, Wirral (3) – Pope
- Charing Cross Hospital & St Mary's Hospital, London (2) – Lewanski/Power
- Wrexham Maelor Hospital (3) – Garcia
- Ysbyty Gwynedd, Bangor (1) – Ghosal

EORTC - 7 Sites

- Ghent University Hospital, Gent, Belgium (15) – Surmont
- Cliniques Universitaires St Luc, Brussels, Belgium (6) – Geets
- Clinique Sainte Elisabeth, Namur, Belgium (5) – Remouchamps
- The Netherlands Cancer Institute, Amsterdam, The Netherlands (4) – Knegjens
- Arnhem S Radiotherapeutisch Institut, Arnhem, The Netherlands (5) – Tissing-Tan
- Medical University, Gdansk, Poland (6) – Jassem
- Institute of Oncology, Ljubljana, Slovenia (13) - Zwitter

SPAIN – 6 Sites

- Institut Català d'Oncologia, Hospital Duran i Reynals, L'Hospitalet (12) – Cardenal
- Institut Català d'Oncologia, Hospital Universitari Germans Trias i Pujol, Badalona (2) – Jove/Carcereny
- University Hospital Ramon y Cajal, Madrid (1) – Hernanz
- Hospital de la Santa Creu i Sant Pau, Barcelona (6) – Majem/Farre
- Institut Oncològic del Vallès (IOV), Barcelona (3) – Solé Monné

- Corporació Sanitària Parc Taulí de Sabadell, Barcelona (3) – Solé Monné/García García

FRANCE – 19 Sites

- Institut de Cancérologie Lucien Neuwirth (2) – Fournel
- Centre Léon Bérard (CLB) (5) – Martel-Lafay
- Hôpital de la Croix-Rousse, Lyon (3) – Arpin
- CHU de Limoges (2) – Vergnenègre/Clavère
- CH de Villefranche/Saône (2) – Falchero
- Centre François Baclesse, Caen (4) – Gervais/Le Rouge
- CH de Aix en Provence (1) – Le Treut
- Institut Ste Catherine, Avignon (12) – Pourel
- CHI de Creteil (6) – Monnet/Martin
- CHU de Clermont-Ferrand (5) – Janicot
- HIA Toulon (1) – Bérard
- Gustave Roussy Cancer Campus, Villejuif (18) – Le Pechoux
- Centre Georges François Leclerc Dijon (5) – Peignaux/Coudert
- Assistance Publique Hôpitaux de Marseille (21) – Padovani/Barlesi
- CHU de Rennes (3) – Lena
- CHU de Caen (2) – Zalcman
- CHU de Lille (6) – Lafitte
- Centre Oscar Lambret, Lille (3) – Prevost
- CH de Libourne (2) – Abdiche

CANADA - 9 Sites

- CHUQ-Pavillon Hotel-Dieu de Quebec, Quebec City (4) – Dagnault
- Cancer Center of SE Ontario at Kingston (5) – de Metz
- Ottawa Hospital Research Institute (13) – Pantarotto
- Princess Margaret Cancer Center, Toronto (7) – Bezjak
- Allan Blair Cancer Center, Regina (2) – Koul
- Saskatoon Cancer Center (1) - Kundapur
- Cancer Care Manitoba (4) – Leylek
- Juravinski Cancer Center (4) – Okawara
- London Regional Cancer Program (1) – Yaremko

Web appendix - Acute adverse events (up to 3 months after completion of study treatment)

AE CTCAE v3·0	Group	0 N (%)	1-2 N (%)	3-5 N (%)	P** Grade 3-5
Nausea	BD<br>OD	70 (26·4)<br>66 (25·1)	172 (64·9)<br>171 (65·1)	23 (8·7)<br>26 (9·9)	0·63
Vomiting	BD<br>OD	146 (55·3)<br>155 (59·0)	105 (39·7)<br>95 (36·1)	13 (4·9)<br>13 (4·9)	0·99
Mucositis	BD<br>OD	173 (65·5)<br>169 (64·5)	88 (33·4)<br>87 (33·2)	3 (1·1)<br>6 (2·3)	0·34
Fatigue	BD<br>OD	21 (8·0)<br>13 (5·0)	212 (80·3)<br>216 (82·4)	31 (11·7)<br>33 (12·6)	0·77
Neuropathy-motor	BD<br>OD	250 (94·3)<br>245 (93·5)	12 (4·5)<br>15 (5·8)	1 (0·4)<br>2 (0·8)	0·62
Neuropathy-sensory	BD<br>OD	196 (74·5)<br>197 (74·9)	63 (24·0)<br>61 (23·2)	4 (1·5)<br>5 (1·9)	>0·99
Infection	BD<br>OD	187 (70·8)<br>181 (69·1)	43 (16·3)<br>52 (19·9)	34 (12·9)<br>29 (11·0)	0·52
Anorexia	BD<br>OD	111 (42·0)<br>112 (42·8)	135 (51·2)<br>129 (49·2)	18 (6·8)<br>21 (8·0)	0·60
Other+	BD<br>OD	37 (14·7)<br>33 (12·6)	150 (57·4)<br>177 (67·6)	76 (28·9)*<br>52 (19·9)	0·02
Haemoglobin	BD<br>OD	39 (14·7)<br>41 (15·7)	194 (72·9)<br>184 (70·8)	33 (12·4)<br>35 (13·5)	0·72
Neutrophils	BD<br>OD	31 (11·7)<br>43 (16·5)	38 (13·7)<br>47 (18·1)	197 (74·1)<br>170 (65·4)	0·03
Febrile neutropenia	BD<br>OD	203 (76·6)<br>214 (82·0)	NA	62 (23·4)<br>47 (18·0) #	0·13
Platelets	BD<br>OD	132 (49·6)<br>119 (45·8)	89 (33·5)<br>87 (33·4)	45 (16·9)<br>54 (20·8)	0·26
Oesophagitis	BD<br>OD	48 (18·9)<br>63 (25·7)	159 (62·6)<br>135 (55·1)	47 (18·5)<br>47 (19·2)	0·85
Pneumonitis	BD<br>OD	198 (78·0)<br>187 (77·3)	51 (20·1)<br>49 (20·2)	5 (2·0)~<br>6 (2·4)~	0·70

BD – twice-daily
OD – once-daily
+ 'Other' grade 3 reported toxicities included:
For OD arm, diarrhoea, hyponatremia, urinary retention and dysphagia and lymphopenia
For BD arm, diarrhoea, constipation, hyponatremia, dysphagia, lymphopenia, dyspnoea and leukopenia
* 2 grade 5 (peripheral arterial ischaemia and 1 septic shock)
# 1 grade 5
~ 3 grade 5 (2 in the once-daily arm & 1 in the twice-daily arm)
**p values are calculated using chi-square tests

Web appendix – Post-Progression Treatment Data

	Twice-daily radiotherapy N=153*	Once-daily radiotherapy N=139*
**Chemotherapy**	88 (58%)	82 (59%)
**Radiotherapy**	42 (27%)	33 (24%)
**Surgery**	8 (5%)	8 (6%)
**Other**	19 (12%)	12 (9%)

*Figures provided from the group of patients who progressed after initial chemo-radiotherapy. The denominators are the number of progressors and the figures are marginal i.e. there may be overlap e.g. chemotherapy + radiotherapy etc.
